# Genetically Obese Human Gut Microbiota Induces Liver Steatosis in Germ-Free Mice Fed on Normal Diet

**DOI:** 10.3389/fmicb.2018.01602

**Published:** 2018-07-20

**Authors:** Ruirui Wang, Hui Li, Xin Yang, Xinhe Xue, Liman Deng, Jian Shen, Menghui Zhang, Liping Zhao, Chenhong Zhang

**Affiliations:** ^1^State Key Laboratory of Microbial Metabolism, School of Life Sciences and Biotechnology, Shanghai Jiao Tong University, Shanghai, China; ^2^Ministry of Education Key Laboratory of Systems Biomedicine, Shanghai Center for Systems Biomedicine, Shanghai Jiao Tong University, Shanghai, China; ^3^Department of Biochemistry and Microbiology, School of Environmental and Biological Sciences, Rutgers New Jersey Institute for Food, Nutrition, and Health, Rutgers University–New Brunswick, New Brunswick, NJ, United States

**Keywords:** gut microbiota, genetic obesity, germ-free mice, liver steatosis, hepatic lipid metabolism

## Abstract

Dysbiotic gut microbiota contributes to genetically obese phenotype in human. However, the effect of genetic obesity-associated gut microbiota on host hepatic metabolic deteriorations remains largely unknown. Gut microbiota from a genetically obese human donor before and after a dietary weight loss program was transplanted into germ-free C57BL/6J male mice, grouped as PreM and PostM groups, respectively. The gut microbiome, liver pathology and transcriptome response in the gnotobiotic mice were evaluated. After being fed on normal chow diet for 4 weeks, PreM group developed liver macrovesicular steatosis accompanied with higher concentrations of hepatic triglyceride and cholesterol, while PostM group exhibited normal hepatic physiology. The gut microbiota in PreM and PostM groups was significantly different from each other and was more resembling with their respective donor. RNA-sequencing revealed that, in comparison with PostM group, PreM group showed a foregoing pro-steatotic transcriptional response in liver featuring by the repression of lipid beta-oxidation and the activation of lipid absorption and cholesterol uptake before the pathology of liver steatosis. Moreover, peroxisome proliferator-activated receptor alpha (PPARα), which was repressed in PreM group, may act as crucial regulator of the hepatic transcriptional profile of lipid metabolism between two groups. Our results show that gut microbiota from a genetically obese human promotes the onset of liver steatosis by impacting hepatic transcriptional profile of lipid metabolism in mice. This adds new evidence that gut microbiota may play a causative role in the development of non-alcoholic fatty liver disease.

## Introduction

Recently, non-alcoholic fatty liver disease (NAFLD) has become one of the most prevalent chronic liver diseases along with the worldwide epidemic of obesity (Bedogni et al., 2005; Younossi et al., 2016). The onset of NAFLD is featured by an excessive accumulation of triglyceride in the cytoplasm of hepatocytes, which brings potentially serious sequelae, such as non-alcoholic steatohepatitis, cirrhosis, and even hepatocellular carcinoma (Petta et al., 2016).

Notably, recent studies have highlighted the pivotal role of gut microbiota in the onset of NAFLD (Bashiardes et al., 2016; Boursier et al., 2016). Fat accumulation in liver induced by diet could be transferred to germ-free (GF) animal through gut microbiota transplantation (Le Roy et al., 2012; Duca et al., 2014). Backhed et al. (2004) has reported that the conventionalization of GF mice could promote the hepatic expression of genes involved in lipid uptake and de novo lipogenesis. Moreover, the persistent activation of genes involved in fatty acid oxidation in liver and skeletal muscle was speculated to be one of the crucial reasons for the resistance to diet-induced obesity in GF mice (Backhed et al., 2007). More recently, Murakami et al. (2016) found that in response to a high-fat diet, gut microbiota could drive the peroxisome proliferator-activated receptor gamma (PPARγ)-mediated activation of hepatic transcriptional circadian oscillation. Therefore, gut microbiota may contribute to the onset of diet-induced obesity and NAFLD through distal regulation of the host hepatic transcriptional response related with lipid metabolism.

In our previous cohort study of children with Prader-Willi syndrome (PWS), one of the most common human genetic diseases leading to obesity (Butler, 2011), we showed that a diet rich in non-digestible carbohydrates could modulate the structure and function of gut microbiota in PWS children and consequently improve their obesity-related phenotypes (Zhang et al., 2015). A human genetic obesity microbiota-associated mouse model was established through conventionalization of GF mice with the gut microbiota from one PWS child before and after diet intervention. We found that the pre-intervention gut microbiota induced a higher level of body fat accumulation and inflammation in the gnotobiotic mice. These results suggest that the dysbiotic, pre-intervention gut microbiota indeed has a greater capacity to induce metabolic deterioration in mice than the post-intervention gut microbiota, implicating a similar role in its PWS hosts (Zhang et al., 2015). However, the effects of gut microbiota associated with genetically predisposed obesity on host hepatic lipid metabolism are still unclear.

In the current study, we conducted an in-depth analysis of hepatic physiology, gut microbiome, and hepatic transcriptome in the human genetic obesity microbiota-associated mouse model. We found that the gut microbiota from a genetically obese child induced liver steatosis in GF mice fed on normal chow diet and revealed the underlying transcriptional process mediated by gut microbiota.

## Materials and Methods

### The Conventionalization of GF Mice

The clinical trial of the PWS cohort (GD58 included) was approved by the Ethics Committee of the School of Life Sciences and Biotechnology, Shanghai Jiao Tong University (No. 2012-016) and registered at Chinese Clinical Trial Registry (No. ChiCTR-ONC-12002646) as described before (Zhang et al., 2015). Written informed consent was obtained from the guardian of the PWS donor (GD58, 8 years old). The procedures and protocols for the animal experiments were approved by Institutional Animal Care and Use Committee of SLAC Inc. Details of the fecal suspension preparation and gut microbiota transplantation of GF male C57BL/6J mice were described previously (Zhang et al., 2015). Mice that received gut microbiota from the PWS human donor pre-intervention (PreH) constituted the PreM group, and mice that received gut microbiota from the PWS human donor post-intervention (PostH) constituted the PostM group. Both PreM (*n* = 10) and PostM (*n* = 9) groups were fed a sterile normal chow diet (D12450B, Research Diets, Inc., United States) in separate plastic isolators for 4 weeks. Feces were collected from each mouse every week. Half of the mice in each group were sacrificed after being colonized for 2 or 4 weeks. Blood, liver, and colon tissues were harvested following refined protocols.

### Liver Histology and Hepatic Lipid Level Assessment

Specific parts of fresh liver from each mouse were fixed in paraformaldehyde for 48 h at room temperature and embedded in paraffin. Five-micrometer-thick sections were stained with hematoxylin and eosin. Images of each liver section were obtained tripartitely by experienced staff who was blinded to the experiment. The liver steatosis score was assessed using Image-Pro Plus, version 7.01 for Windows (Media Cybernetics, Silver Springs, MD, United States) as described previously (Catta-Preta et al., 2011).

A frozen pellet of liver sample was homogenized in a corresponding volume (W/V:1/9) of homogenizing buffer (pH 7.4, 0.01 mol/L Tris-HCl, 1 mmol/L EDTA, 0.8% NaCl). The supernatant was collected after being centrifuged at 2000 g for 25 min at 4°C. Assay kits (NanJing Jiancheng Bioengineering Institute, China) were used to measure the hepatic and serum triglyceride and cholesterol concentrations. The results of hepatic lipids were corrected for total protein concentration (Yeasen Biotechnology Co., Ltd., China).

### Enzyme-Linked Immunosorbent Assay

Enzyme-linked immunosorbent assay kits were used to determine the serum concentration of leptin (R&D Systems, United States), lipopolysaccharide (LPS)-binding protein (LBP) (Cell Sciences, United States), and serum amyloid A protein (SAA, Tridelta, Ireland) according to the manufacturer’s instructions.

### Gut Microbiota Profiling

The bacterial DNA from feces of the human donor and mouse recipients after transplantation for 1, 2, 3, and 4 weeks was extracted as previously described (Godon et al., 1997) and checked for concentration and purity (Qubit2.0, Thermo Fisher). The variable region 3 (V3) of bacterial 16S rRNA gene in all samples from the donor and recipients were sequenced in the same run by Ion Torrent^TM^ Next-Generation Sequencing (Thermo Fisher). The barcode library was prepared following the Ion Torrent PGM protocol for short amplicons (<350 bp). The primers were composed of a universal primer (341F: 5′ CCTACGGGAGGCAGCAG 3′, 518R: 5′ ATTACCGCGGCTGCTGG 3′) for the bacterial 16s rRNA gene V3 region, a sample-specific barcode, and a PGM sequencing adaptor. PCR was conducted using amplification conditions as described previously (Milani et al., 2013). After checking for size and concentration, the purified PCR product of each sample was mixed equally. The mixture was then pretreated on Ion OneTouch^TM^ using Ion PGM Template OT2 200 Kit and sequenced on PGM system using Ion 314 Chip Kit v2.

The raw reads were pretreated as described previously (Milani et al., 2013). High-quality reads were then assigned to closed reference operational taxonomic units (OTUs) at 97% similarity levels following the procedure in QIIME platform (version 1.8) (Caporaso et al., 2010). Using the rarefaction OTU table, we performed principal coordinate analysis (PCoA) based on weighted UniFrac distances. Multivariate analysis of variance using PCoA accounting for 80% of total variations was conducted in R version 3.4.0^1^. The alpha diversity of each sample was calculated based on the number of observed OTUs and the Shannon index.

Considering the stability of the gut microbiota in each group of recipient mice over time, all time points in different groups were merged to identify discriminating OTUs between two groups by a random forest model through leave-one-out cross-validation (Breiman, 2001). A visually selected cut-off value for discriminating OTUs was chosen according to the descending accuracy of discrimination of all the OTUs. The abundance variations in OTUs between different groups in donor (PostH divided by PreH) and recipients (PostM divided by PreM) were assessed. A heat map of the key OTUs was generated in R version 3.4.0 (see footnote 1), with the abundance of the OTUs which was log2-transformed.

### Hepatic Transcriptome Profiling

Total RNA in mice livers was extracted using the RNeasy mini kit (Qiagen, Germany). After purification using the RNase-Free DNase Set (Qiagen), the concentration and quality of the purified RNA were determined using the NanoDrop ND-1000 (Thermo Scientific, United States) and Agilent Bioanalyzer 2100 systems (Agilent Technologies, United States). Libraries for sequencing were generated according to the Ilumina TrueSeq protocol. Paired*-*end RNA sequencing was performed on an Illummina HiSeq 2500 according to the manufacturer’s instructions with a quality control standard (each sample sequencing depth higher than 7 GB, read length higher than 90 nt, and Q30 > 85%).

The raw RNA-seq reads in FASTQ format were processed to trim the adaptor using Flexbar (Dodt et al., 2012), and the reads containing ploy-N and the low-quality reads were removed using Prinseq (Schmieder and Edwards, 2011). The high-quality reads from each sample were aligned to the Mus musculus reference genome, GRCm38 (Genome Reference Consortium mouse build 38^2^), using HISAT (Hierarchical Indexing for Spliced Alignment of Transcripts, version 0.1.7-beta) with default parameters (Kim et al., 2015). The unambiguous alignments of each sample were quantified by HTSeq-count (version 0.6.1p1) (Anders et al., 2015) with the mouse genome annotation GTF file^3^, and the gene expression profile in terms of the read counts was acquired. After obtaining the gene counts of each sample, differential expression analysis was performed using DESeq2 (version 1.9.23) (Love et al., 2014). Differentially expressed genes (DEGs) between PreM and PostM groups were determined by Deseq2 with the standard of |log2FoldChange|≥ 1 on each time point. The normalized gene count matrix constructed by DESeq2 was extracted for further statistical analysis. Hierarchical clustering (spearman correlation) and expression visualization of all the DEGs were performed in R version 3.4.0, with the abundance of the DEGs transformed by *z*-score (standard score).The DEGs were analyzed using Ingenuity Pathway Analysis (IPA, QIAGEN^4^) for further functional analysis. Fisher’s exact right-tailed test identified statistically significantly different [-log(*P*-value) ≥ 1.3] pathways or biological functions. Taken the change fold and direction of DEGs into account, an IPA *z*-score was computed to determine whether the pathway or biological functions were significantly activated or inhibited (|*z*-score|≥ 2). Network analysis was performed with selected biological functions and related genes to identify the crucial regulators.

### Quantitative Real-Time PCR

Total RNA isolated from the liver and colon was reverse-transcribed to cDNA using a SuperScriptTM first-strand synthesis system for RT-PCR (Invitrogen, United States). Quantitative real-time PCR (qRT-PCR) was performed on a LightCycler96 (Roche, Switzerland) using iQ SYBR Green Supermix (BIO-RAD, United States). The relative expression of *Pparα, Pparγ, Alt* (gene encode alanine aminotransferase), and *Ast* (gene encode aspartate aminotransferase) were adjusted with Glyceraldehyde-3-phosphate dehydrogenase (*Gapdh*) as the housekeeping gene and then normalized to that in PreM group on the 2^nd^ week. The primer sequences for qRT-PCR were as follows:

*Pparα* (F: 5′ AACATCGAGTGTCGAATATGTGG 3′, R: 5′ AGCCGAATAGTTCGCCGAAAG 3′);

*Pparγ* (F: 5′ TCGCTGATGCACTGCCTATG 3′, R: 5′ GAGAGGTCCACAGAGCTGATT 3′);

*Alt* (F: 5′ CTGCAGACCCGAACAACATA 3′, R: 5′ CAGCTCAGCGATGTCAAGAG 3′);

*Ast* (F: 5′ GCTGACTTCTTAGGGCGATG 3′, R: 5′ TCATTCAGGAAACCCTGGAG 3′);

*Gapdh* (F: 5′ GTGTTCCTACCCCCAATGTGT 3′, R: 5′ ATTGTCATACCAGGAAATGAGCTT 3′).

### Western Blot Analysis

Frozen pellets of liver and colon sample were homogenized in modified RIPA buffer (50 mmol/L Tris Base, 1% TritonX-100, 0.5% sodium deoxycholate, 0.1% sodium dodecyl sulfate (SDS), phosphatase inhibitor cocktail (Biotool, Houston, TX, United States), and protease inhibitor cocktail (Biotool, Houston, TX, United States). After centrifugation at 2000 ×*g* for 25 min at 4°C, the resulting supernatant was collected. Aliquots (50,000 ng of protein) of the protein were separated through SDS-polyacrylamide gel electrophoresis and transferred to nitrocellulose filter membranes (ExCell Bio, Shanghai, China). Membranes were blocked with 5% (w/v) bull serum albumin (BSA; MP Biomedicals New Zealand Limited, New Zealand) in Tris-buffered saline containing 0.1% Tween 20 (TBS-T) and incubated with antibody in TBS-T containing 5% BSA and anti-rabbit PPARα or β-ACTIN antibody (1:1000; Abcam, Cambridge, United Kingdom). After incubation overnight, the membranes were washed with TBS-T and then incubated with anti-rabbit IgG secondary antibody (Gibco BRL, Gaithersburg, MD, United States) in 5% BSA in TBS-T. After the membranes were washed, the immune complexes were detected using a chemiluminescence system (LI-COR, Lincoln, NE, United States). Then PPARα immunoblots were quantified by densitometry analysis using ImageJ software against β-ACTIN as internal controls.

### Immunohistochemistry

After deparaffinized and processed for antigen retrieval, the blank liver sections were incubated with anti-rabbit PPARα antibody (1:1000; Abcam, United States) for 37°C for 2 h. Then incubated with anti-rabbit IgG secondary antibody (KPL, Massachusetts, United States) for 30 min at room temperature. After washed by PBS, DAB Substrate kit (Solarbio, Beijing, China) was used to develop color. Images of each liver section were obtained tripartitely by experienced staff who was blinded to the experiment under 100 times magnification using Leica DMRBE microscope.

### Statistical Analysis

Differences in the biochemical index and the distance of the gut microbiota between groups were assessed by the Mann-Whitney *U* test using GraphPad Prism 7 software. *P* < 0.05 was used to indicate significant differences. Other statistical analyses are described in the corresponding results section.

### Sequence Data Accession Numbers

The 16S rRNA gene sequencing and hepatic transcriptome data have been submitted to the GenBank Sequence Read Archive database, with accession numbers of SRP132268 and SRP132325, respectively.

## Results

During our previous dietary intervention program of genetically obese children with PWS, we selected an eight-year-old boy (GD58) as the representative of the cohort for the subsequent gut microbiota transplantation experiment (Zhang et al., 2015). During the dietary intervention, this child showed substantial improvement of NAFLD and liver function, along with systemic alleviation of other obesity-related phenotypes (Supplementary Table S1). Meanwhile, the shift of the gut microbiota in this PWS child was concordant with that in the PWS cohort during the dietary intervention (Supplementary Figure S1). The fecal samples from this PWS donor were collected before (PreH) and after (PostH) dietary intervention and transplanted into GF mice which were named PreM and PostM groups, respectively.

### Pre-intervention Gut Microbiota Induces Hepatic Steatosis in Recipient Mice Fed on Normal Chow Diet

**FIGURE 1:**
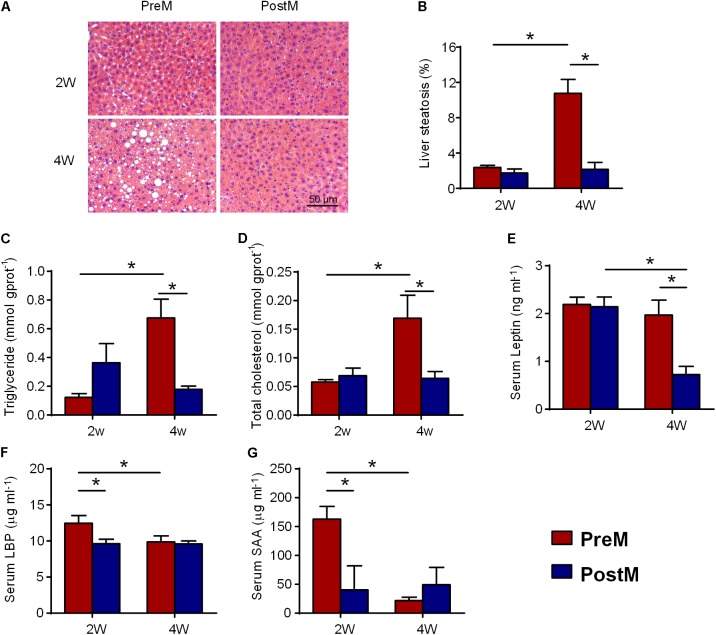
PreM group showed increased lipid concentration and fat accumulation in liver after transplantation for 4 weeks; **(A)** Representative photomicrographs of hematoxylin- and eosin-stained sections of the liver (100× magnification); **(B)** bar graph of the volume density of liver steatosis; **(C)** hepatic triglyceride; **(D)** hepatic total cholesterol; **(E)** serum leptin; **(F)** serum LBP; and **(G)** serum SAA levels. Data are shown as the mean ± SEM and were compared between groups and time points using the Mann–Whitney *U* test; ^∗^*P* < 0.05. *n* = 4 or 5 for each group. LBP, lipopolysaccharide binding protein; SAA, serum amyloid A protein.

To evaluate the effect of human genetic obesity-associated gut microbiota on host hepatic lipid metabolism, we assessed the lipid concentration and fat accumulation in the liver of the gnotobiotic mice. Hematoxylin and eosin staining of mouse liver sections showed that both groups exhibited normal liver histology 2 weeks after transplantation (**Figures 1A,B**). However, PreM group showed a higher level of serum LBP on the 2^nd^ week (**Figure 1F**), reflecting a higher level of endotoxemia. The increased SAA on the 2^nd^ week (**Figure 1G**) also suggested a higher level of systemic inflammation in PreM group compared with PostM group. During the following 2 weeks, PreM group developed liver macrovesicular steatosis, while PostM group remained normal morphology (**Figure 1A**). The steatosis score was significantly higher in PreM group than that in PostM group on the 4^th^ week (**Figure 1B**). Accordingly, the concentrations of hepatic triglyceride and total cholesterol of PreM group were significantly higher than PostM group on the 4^th^ week (**Figures 1C,D**), so is the serum triglyceride (Supplementary Figure S2). The expression of *Ast*, which encode aspartate aminotransferase, was significantly higher in PreM group compared to PostM group on the 4^th^ week, while the expression of *Alt*, which encode alanine aminotransferase, showed no difference between groups (Supplementary Figure S3), indicating the heavier liver function in PreM group. Meanwhile, the concentration of leptin, which is an adipocytokine, was also significantly higher in PreM group on the 4^th^ week (**Figure 1E**). These results suggested that the pre-intervention gut microbiota induced a higher level of bacterial stimuli and subsequently increased hepatic lipid concentration and accumulation in gnotobiotic mice.

### The Gut Microbiota of Recipient Mice Was More Similar to Their Respective Donor

**FIGURE 2:**
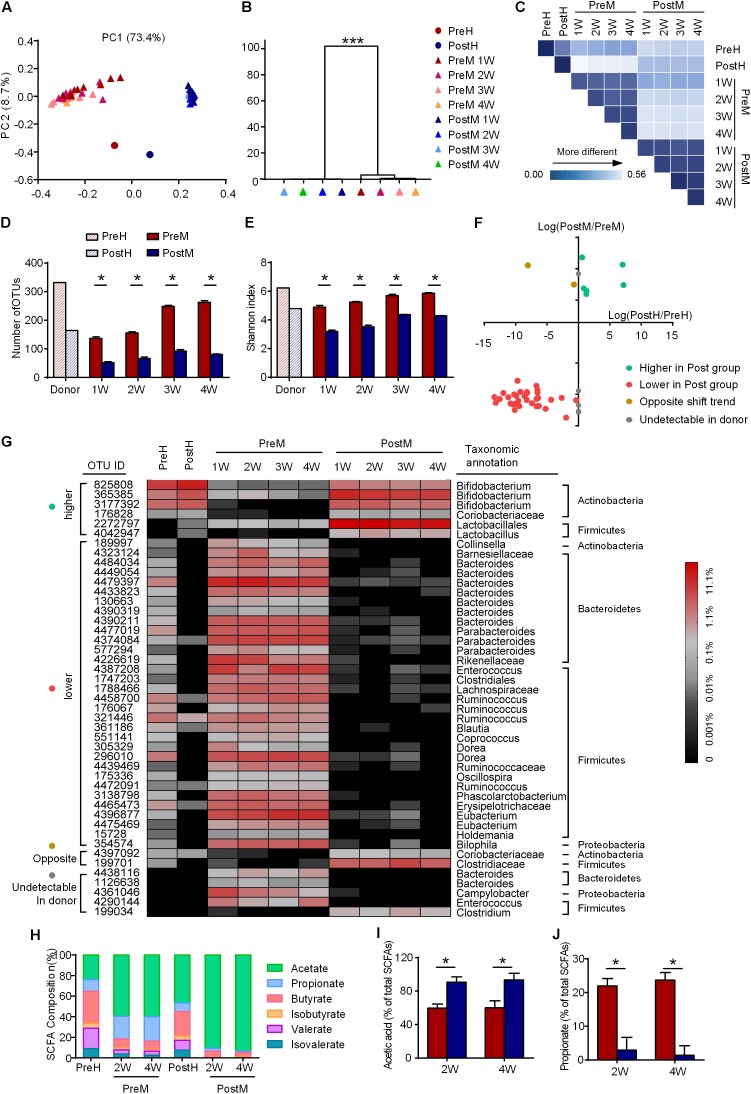
The features of gut microbiota are transferrable from the human donor to the GF mice. **(A)** Weighted UniFrac PCoA plot and **(B)** multivariate analysis of variance analysis of gut microbiota based on OTUs, ^∗∗∗^*P* < 0.001; **(C)** weighted UniFrac distances of gut microbiota between the donor and recipients; **(D)** OTU richness; **(E)** Shannon index; **(F)** abundance variation of 45 discriminatory OTUs in the donor and recipients; **(G)** heat map of 45 discriminatory OTUs, with the color of the spot corresponding to the normalized and log-transformed relative abundance; **(H)** the composition of SCFA in feces of donor and the cecum of the recipients; **(I)** relative concentrations of acetate; and **(J)** relative concentrations of propionate. For the recipient groups, *n* = 9 on the 1^st^ and 2^nd^ week; *n* = 4 or 5 on the 3^rd^ and 4^th^ week. In **D,E,I**, and **J**, data are shown as the mean ± SEM and were compared between groups using the Mann–Whitney *U* test. ^∗^*P* < 0.05. PC, principal coordinate; OTU, operational taxonomic unit; SCFA, short chain fatty acid.

The gut microbiota of the human donor (PreH and PostH) and mice recipients (PreM and PostM) were profiled by sequencing of the bacterial 16S rRNA gene V3 region on Ion Torrent PGM System. On average, each sample obtained 20,727 ± 5173 high-quality reads. The high-quality reads were then delineated into 1123 OTUs at the similarity cutoff of 97% (Supplementary Table S2). PCoA analysis of weighted UniFrac distances based on OTUs revealed a clear separation of gut microbiota between PreM and PostM groups as well as the PreH and PostH groups on the first principal coordinate (PC1, accounting for 73.4% of the total variance) (**Figure 2A**). Multivariate analysis of variance analysis derived from PCoA scores confirmed a statistically significant (*p*-value < 0.001) separation of PreM and PostM group. Gut microbiota in each group showed little changes over time, indicating the gut microbiota kept stable after transplantation (**Figure 2B**). The gut microbiota of the donor and recipients were mainly distributed on PC2 (accounting for 8.7% of the total variance), reflecting the selectivity of the microbiota during the transplantation from the human to the mice (**Figure 2A**). The weighted UniFrac distance between the gut microbiota of the donor and recipients further revealed that each recipient group was more similar to their own donor (**Figure 2C** and Supplementary Figure S4). Nevertheless, decreased diversity and richness of gut microbiota in the PostH group were also recurred in PostM group, as measured by the Shannon index and the number of OTUs (**Figures 2D,E**).

Using a random forest model, 45 OTUs were picked out as discriminating features between PreM and PostM groups (Supplementary Figure S5 and Supplementary Table S2). These key OTUs accounts for 52.8% of the abundance of all the OTUs. Through the abundance pairwise comparisons of these OTUs between the donor (axis *X*) and recipients (axis *Y*) (**Figure 2F**), 38 OTUs showed consistent abundance variation between different groups in the donor and recipients. Six OTUs in the first quartile, including OTUs from *Bifidobacterium* and *Lactobacillus*, were less abundant in PreM group as in PreH. 32 OTUs in the third quartile were more abundant in PreM group as in PreH, while almost diminished in PostM group as in PostH, which are primarily belonged to Bacteroidetes (e.g., *Bacteroides* and *Parabacteroides*) and Firmicutes (e.g., *Ruminococcus*), as well as one belonged to Proteobacteria (*Bilophila*) (**Figures 2F,G**).

Meanwhile, the microbial fermentation products, short-chain fatty acids (SCFAs), showed a lower ratio of acetate and a higher ratio of propionate in PreM group as in PreH donor, compared with PostM group and PostH donor (**Figures 2H–J**). So, on the whole structure, bacteria species and bacterial product level of the gut microbiota, our results showed that the major features of the gut microbiota were transferrable from the PWS donor to the recipient mice.

### Pre-intervention Gut Microbiota Induces a Steatosis-Prone Transcriptional Program

**FIGURE 3:**
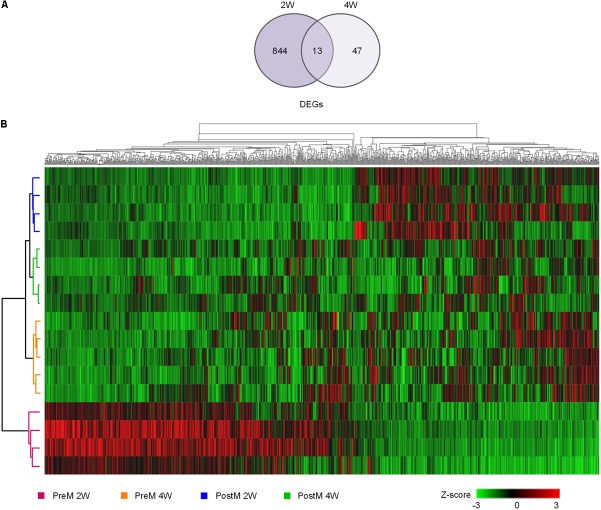
The expression pattern of DEGs in the liver of gnotobiotic mice over time. **(A)** Venn diagrams of the DEGs between the two groups on week two and week four determined by Deseq2 with the standard of |log2FoldChange|≥ 1; **(B)** hierarchical clustering of all the DEGs, with the color of the spot corresponding to the normalized and *z*-score transformed relative abundance. *n* = 4 or 5 for each group. DEGs, differentially expressed genes.

To explore the biological process and mechanism underlying the development of liver steatosis mediated by gut microbiota, the hepatic transcriptional response in the recipient mice was monitored by mRNA sequencing on the 2^nd^ and 4^th^ week after transplantation. Deseq2 was used to identify DEGs between the two groups (|Log2(foldchange)|> 1). In total, 857 DEGs were identified on the 2^nd^ week, while only 60 DEGs were identified on the 4^th^ week (**Figure 3A**). All the DEGs (904 genes in total) were hierarchically clustered and revealed that hepatic gene expression patterns in PreM group on the 2^nd^ week were distinct from those of other groups (**Figure 3B**), indicating that pre-intervention gut microbiota induced a specific disturbance on the transcriptional level in the liver of gnotobiotic mice after colonization for 2 weeks.

We then interpreted the data using IPA (QIAGEN see footnote 4) based on the comprehensive, manually curated content of the Ingenuity Knowledge Base. All the DEGs were input into the IPA software to identify statistically significantly different (calculated by Fisher’s exact test, right-tailed, *P*-value < 0.05) and biologically significantly modulated (using an algorithm to predict the direction of change, *z*-score ≥ 2 for suppression and *z*-score ≤-2 for activation in PreM compared to PostM group) biological functions and pathways between the two groups.

**FIGURE 4:**
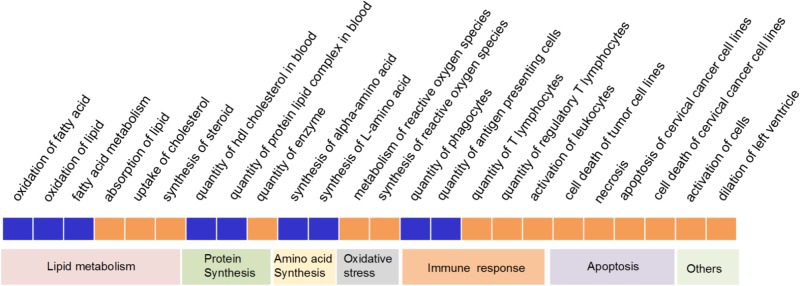
Significantly repressed or activated functions in the liver of gnotobiotic mice on the 2^nd^ week based on IPA. Blue represent significantly repressed functions (*z*-score ≥ 2), while red represent significantly activated (*z*-score ≤ –2) functions in PreM group compared to PostM group.

On the 2^nd^ week, over 500 biological functions mainly related with small molecule biochemistry, lipid metabolism and molecular transport were significantly enriched (Supplementary Table S3), therein, 24 of which showed a significantly activated or suppressed state between PreM and PostM groups (**Figure 4**). Most notably, in PreM group, functions related to lipid catabolism including oxidation of fatty acid, oxidation of lipid and fatty acid metabolism were significantly repressed. While functions related to lipid anabolism, including absorption of lipid, uptake of cholesterol and synthesis of steroid were significantly activated. Two functions related to lipid transport, i.e., quantity of HDL cholesterol and protein lipid complex in blood, were also significantly repressed by the pre-intervention gut microbiota. The functions of alpha-amino acid and L-amino acid synthesis were significantly repressed in PreM group reflecting a decreased level of amino acid anabolism. Meanwhile, functions related to oxidative stress and apoptosis were significantly activated in PreM group. Additionally, the functions related to adaptive immune responses (i.e., biological function “quantity of T lymphocytes and regulatory T lymphocytes”) were also significantly activated in PreM group.

We further identified 71 pathways that were significantly enriched between PreM and PostM group on the second week using IPA (Supplementary Table S4). These pathways were involved in various biological processes, including metabolism, signal transduction, and immune response. Of these 71 pathways, four were significantly activated or repressed between the two groups (**Table 1**). The PPARα/RXRα activation pathway was significantly suppressed in PreM group. Meanwhile, the PI3K/AKT signaling pathway and two pathways related to coagulation (extrinsic prothrombin activation and coagulation system pathway) were significantly activated in PreM group.

**Table 1:** Significantly repressed or activated pathways in liver between the two groups on the second week.

No.	Ingenuity Canonical Pathways	*z*-score^∗^	‒log (*P*-value)	Molecules
1	PPARα/RXRα activation	2	1.73	10
2	Extrinsic prothrombin activation	−2	3.20	4
3	Coagulation system	−2.24	2.71	5
4	PI3K/AKT signaling	−2.45	1.38	7

*^∗^z-scores ≥ 2 represent significantly repressed functions, while z-scores ≤−2 represent significantly activated functions in PreM group compared to PostM group.*

No function or pathway was significantly repressed or activated between the two groups 4 weeks after transplantation, indicating that the difference on the transcriptional level between PreM and PostM groups got diminished along with time. Taken together, the results indicate that before the pathology of steatosis was diagnosed, the pre-intervention gut microbiota induced a foregoing pro-steatotic transcriptional pattern in the liver of gnotobiotic mice.

### PPARα May Play a Crucial Role in Regulating Hepatic Transcriptional Profile of Lipid Metabolism in Gnotobiotic Mice

**FIGURE 5:**
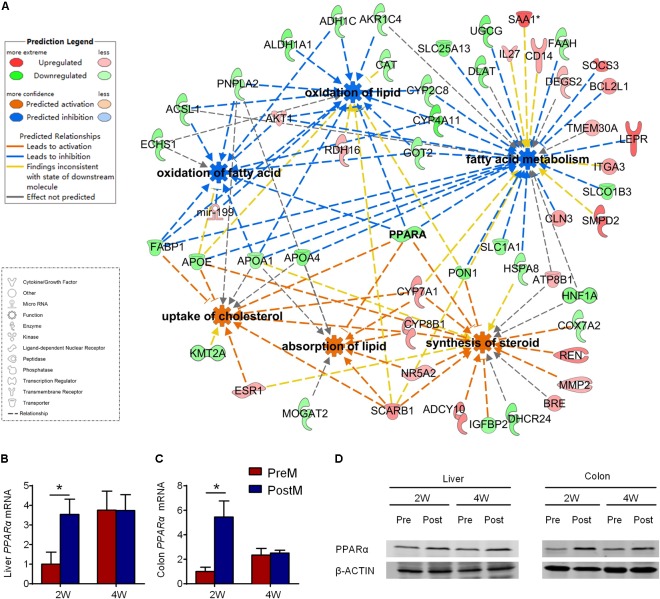
The hepatic transcriptional profile of lipid metabolism in gnotobiotic mice. **(A)** IPA ingenuity interaction network of six significantly modulated biological functions (circle with eight arises) and 53 DEGs (configuration corresponding to the type of gene product as showed in the lower figure legend) related to lipid metabolism in liver on the 2^nd^ week. As showed in the upper legend, DEGs with red color represent for upregulated while green color represent for downregulated in PreM group compared to PostM group, biological functions with blue color are predicted to be significantly repressed, while orange color are predicted to be significantly activated in PreM group compared to PostM group. Lines represent the relationship between the biological functions and DEGs. **(B)** RT-PCR of *pparα* expression in liver. **(C)** RT-PCR of *pparα* expression in colon. The relative expression of *Pparα* was adjusted with *Gapdh* as the housekeeping gene. **(D)** Western blot of PPARα and β-ACTIN in liver and colon. Data are shown as the mean ± SEM and were analyzed by the Mann–Whitney *U* test between groups; ^∗^*P* < 0.05. *n* = 4 or 5 for each group. The details of the DEGs refer to Supplementary Table S5.

The six significantly modulated lipid metabolism functions associated with 53 DEGs, which all differed on the 2^nd^ week, and only one (fatty acid binding protein 1, FABP1) remained different until the 4^th^ week (Supplementary Table S5). We then profiled the lipid metabolism network of the functions and DEGs using IPA (**Figure 5A**). Genes encoding crucial enzymes in lipid catabolism, especially lipid beta-oxidation, were down-regulated in PreM group, such as patatin-like phospholipase domain containing 2 (*Pnpla2*), acyl-CoA synthetase long-chain family member 1 (*Acsl1*) and enoyl CoA hydratase, short chain, 1 (*Echs1*). Meanwhile, the expression of genes involved in lipid binding and transport, such as fatty acid binding protein 1 (*Fabp1*), apolipoprotein A1 (*Apoa1*), apolipoprotein A4 (*Apoa4*), and apolipoprotein E (*Apoe*), was also decreased in PreM group. In contrast, genes involved in lipid absorption, steroids, and cholesterol metabolism, such as cytochrome P450 family 7 subfamily A member 1 (*Cyp7a1*), cytochrome P450 family 8 subfamily B member 1 (*Cyp8b1*), scavenger receptor class B member 1 (*Scarb1*), and adenylate cyclase type 10 (*Adcy10*), were up-regulated in PreM group (**Figure 5A**).

Notably, PPARα contributed to all the significantly modulated functions in the network (**Figure 5A**). PPARα is a transcriptional factor that plays a crucial role in lipid metabolism by directly inducing the expression of downstream genes (Pawlak et al., 2015). Seventeen of the 53 DEGs in the lipid metabolism network could be targeted by PPARα (Supplementary Figure S6). The suppression of *Pparα* contributed to the suppression of genes related to lipid catabolism, such as *Acsl1, Apoa1, Apoa4, Apoae*, and *Fabp1*, and the activation of *Cyp7a1* as predicted in the network. As verified by quantitative real-time PCR, the expression of *Ppar*α was significantly suppressed not only in the liver but also in the colon of PreM group on the 2^nd^ week compared to PostM group (**Figures 5B,C**). Western blot and immunohistochemical analysis also indicated a suppression of PPARα on protein level in PreM group compared to PostM group (**Figure 5D** and Supplementary Figures S7 and S8). Whereas, the expression of another key modulator of lipid metabolism in PPARs family – *Pparγ* showed no difference between two group on both 2^nd^ and 4^th^ week (Supplementary Figure S9). Our results indicates that the suppression of PPARα may play a regulating role contributing to the pro-steatotic transcriptional profile in PreM group.

## Discussion

In previous study, we showed that genetically obese children with PWS showed significantly improved liver functions after they have lost substantial amount of body weight on a dietary program targeting the gut microbiota [11]. In the current study, we showed that the baseline (pre-intervention) gut microbiota from one genetically obese child with PWS induced liver steatosis in GF mice fed on normal chow diet, while the gut microbiota from the same donor after the dietary intervention did not induce this early form of NAFLD.

Germ-free mice is considered as a powerful animal model to demonstrate the causal role of gut microbiota in the development of many metabolic diseases (Turnbaugh et al., 2009; Faith et al., 2014). Previous studies usually studied the contribution of gut microbiota to human disease using gnotobiotic animal model established through the conventionalization of GF animal by gut microbiota from disease or healthy control individuals (Ridaura et al., 2013; Llopis et al., 2016; Nagao-Kitamoto et al., 2016). However, the configurations of gut microbiota which could be affected by genetic and environmental variables vary substantially among individuals (Human Microbiome Project Consortium, 2012), which may confound the phenotype-associated microbial features. In our study, GF mice were conventionalized by gut microbiota from one PWS child before and after a dietary intervention, which significantly alleviated his obesity-related phenotypes. The comparison of gnotobiotic mice associated with gut microbiota from the same individual but having a distinct metabolic phenotype could minimize the individual-related variations, therefore, providing an opportunity to reveal the interaction between the host and gut microbiota which is more relevant with the metabolic phenotypes. More importantly, the liver steatosis observed in the GF mice receiving baseline gut microbiota from the PWS child was independent of diet, the major environmental factor to induce NAFLD. In this case, the baseline pre-intervention gut microbiota from the PWS donor is the only driver of liver steatosis in the gnotobiotic mice.

Microbiome-wide association studies make it possible to discover the disease-modulating microbes (Qin et al., 2010). Recently, Surana and Kasper (2017) proposed a microbe-phenotype triangulation to narrow the scope of the causal microbes, which is persistently correlated with defined phenotypes under different conditions. Given the reproducibility of the obesity-related phenotype in GF mice from the human PWS donor, the transferrable configurations of gut microbiota have a high probability acting as contributors causally associated with the metabolic phenotype in mice, which could also be implicated in the PWS donor. Through the metagenomics analysis in our previous cohort study, we found that co-abundance gene groups identified as *Bacteroides, Parabacteroides*, and *Ruminococcus* were abundant in the PWS cohort before diet intervention and regarded as potential pathogens on account of their genetic potential to produce toxic co-metabolites such as trimethylamine N-oxide and indoxyl sulfate (Zhang et al., 2015). The higher abundance of these OTUs may have exerted the same deteriorating effect in PreM group. Another abundant OTU in PreM group belongs to *Bilophila*, an opportunistic pathogen that has been correlated with many human inflammatory diseases (Baron et al., 1989; Feng et al., 2017). Bacteria from *Bifidobacterium* and *Lactobacillus* are generally regarded as beneficial, which have been reported having the capacity to attenuate weight gain and hepatic steatosis (Reichold et al., 2014; Wang et al., 2015). Furthermore, *Bifidobacterium* and its fermented product acetate have been reported having an inhibitory effect on many species of detrimental bacteria (Lievin et al., 2000; Fooks and Gibson, 2002). The higher level of the relative concentration of acetate among SCFAs and *Bifidobacterium* may have contributed to the decrease of the potential pathogenic bacteria in PostM group. These bacteria are highly indicated as functional bacteria relevant with host metabolic phenotypes. Functional validation and the underlying mechanism of these phenotype-associated bacteria should be further investigated.

The conventionalization of GF mice has been reported to induce metabolic reorientation and immune responses by process in both liver and intestine (Claus et al., 2011; El Aidy et al., 2012, 2013). PPARα serves as a crucial regulator of lipid metabolism in liver, while its deficiency can induce liver steatosis (Akiyama et al., 2001; Kersten and Stienstra, 2017). GF mice showed significantly higher expression of PPARα in the liver and intestine compared to conventionalized mice, indicating the expression of PPARα in peripheral tissues could be affected by the commensal bacteria in gut (El Aidy et al., 2013; Selwyn et al., 2015). Notably, LPS, a constituent of the outer membrane of gram-negative bacteria, exhibits the capability to repress the hepatic expression of PPARα (Beigneux et al., 2000). In our study, the increased level of serum LBP and the higher hepatic expression of the membrane receptors of LPS, *Cd14* and *Tlr4* (Zhang et al., 2015), reflected a higher level of serum LPS in PreM group, which may have contributed to the suppression of PPARα in liver. On the contrary, prebiotic or probiotic treatment could increase the PPARα expression in liver (Pachikian et al., 2013; Mei et al., 2015). Meanwhile, acetate, the product of many probiotics, may significantly increase the hepatic expression of PPARα and its downstream genes related to fatty acid oxidation (Kondo et al., 2009). In the current study, the acetate concentration and the abundance of its producer were both significantly higher in PostM group. These results indicate that the suppression of hepatic expression of PPARα by pre-intervention gut microbiota may be due to the increased production of LPS and the decreased production of acetate. Our results also indicated a time-resolved hepatic transcriptional response mediated by gut microbiota and a delayed development “catching up” of liver steatosis. This temporal order of events from microbiota-induced gene expression changes in liver lipid metabolism to the actual manifestation of liver steatosis strongly implicates a causative role of gut microbiota in NAFLD.

## Summary

The effect of gut microbiota in the development of obesity and NAFLD has been widely realized. The current study show that the gut microbiota from a genetically obese human could promote the onset of liver steatosis in mice independent from diet and genetic factors. Our result also give a comprehensive profile of hepatic lipid metabolism and suggested that PPARα may be the key regulator in the gut microbiota induced hepatic steatosis. The plasticity of gut microbiota makes it a potentially preventive and therapeutic target of NAFLD and other metabolic diseases.

## Author Contributions

RW, CZ, and LZ conceived the project, designed the experiments, and wrote the manuscript. RW, XY, and XX performed and analyzed the animal experiments. RW carried out the gut microbiome analysis. HL, RW, LD, and MZ established the RNA sequencing analysis pipeline and carried out the analysis. RW and JS prepared the fecal suspension for the transplantation experiment. All authors commented on and approved the manuscript.

## Conflict of Interest Statement

The authors declare that the research was conducted in the absence of any commercial or financial relationships that could be construed as a potential conflict of interest.

## Footnotes

1 https://www.r-project.org/

2 ftp://ftp.ensembl.org/pub/release-80/fasta/mus_musculus/dna/

3 ftp://ftp.ensembl.org/pub/release-80/gtf/mus_musculus

4 http://www.ingenuity.com
