# Supplementary material for: Genetically Obese Human Gut Microbiota Induces Liver Steatosis in Germ-Free Mice Fed on Normal Diet
Source: Front Microbiol. 2018 Jul 20;9:1602. doi: 10.3389/fmicb.2018.01602 (PMC6062601; doi:10.3389/fmicb.2018.01602)
Supplement: Supplementary file 1 [file Data_Sheet_1.PDF]

Supplementary Figures and Tables

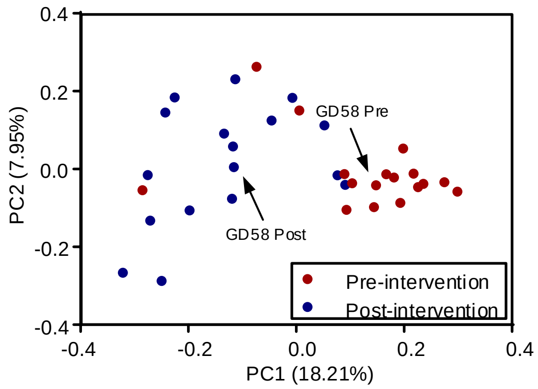

Figure S1 The structural shift of gut microbiota of GD58 was concordant with the whole cohort (n=17) as revealed by weighted UniFrac Pcoa plot.

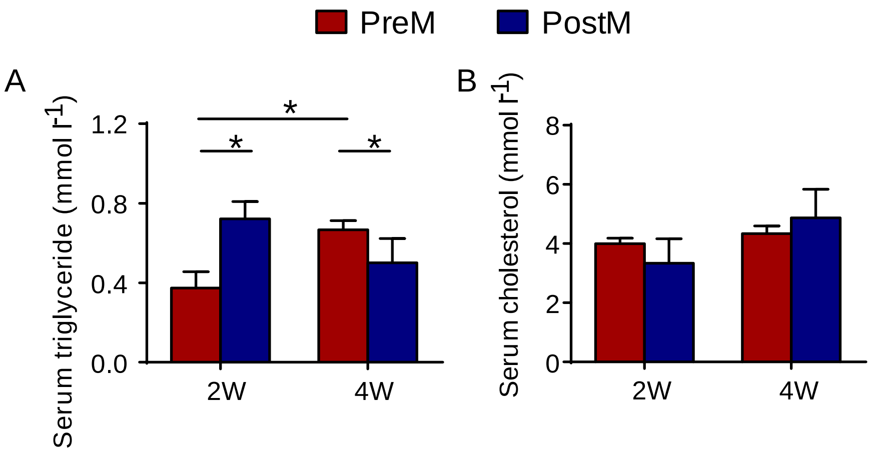

Figure S2 The serum triglyceride (A) and total cholesterol concentration (B) in gnotobiotic mice. Data are shown as the mean±s.e.m. and were compared between groups and time points using the Mann-Whitney U test. *P<0.05. n=4 or 5 for each group.

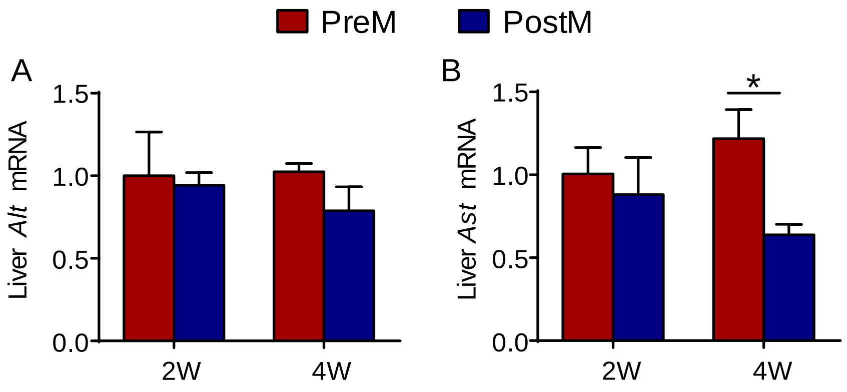

Figure S3 The expression of *Alt* (A) and *Ast* (B) in liver of gnotobiotic mice. The relative expression of *Alt* and *Ast* were adjusted with *Gapdh* as the housekeeping gene. Data are shown as the mean±s.e.m. and were compared between groups using the Mann-Whitney U test. *$P<0.05$. n=4 or 5 for each group. *Alt*, gene encode alanine aminotransferase; *Ast*, gene encode aspartate aminotransferase.

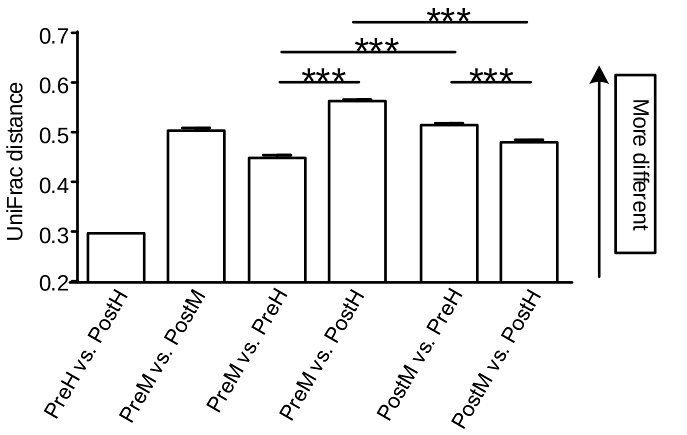

Figure S4 Weighted UniFrac distances of gut microbiota between human donor and mice recipients. Data are shown as means±s.e.m. and test by Mann–Whitney U test between groups. ***$P < 0.0001$,

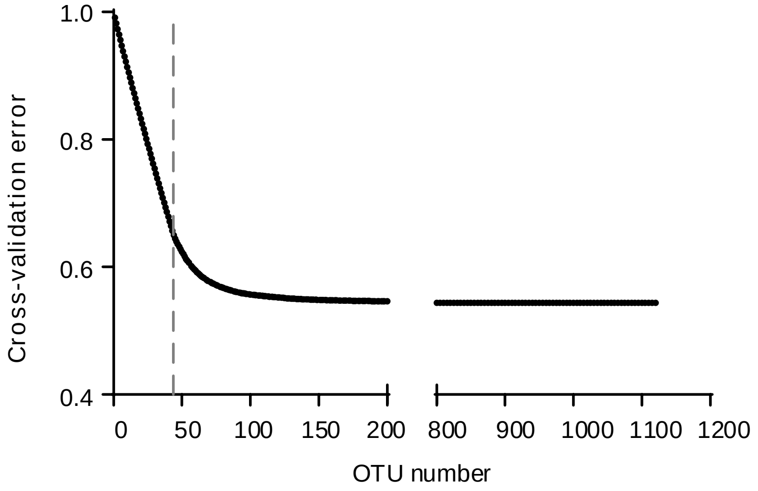

Figure S5 45 key OTUs were identified as key OTUs by random forest classification, ranking by descending order of their feature accuracy for the models.

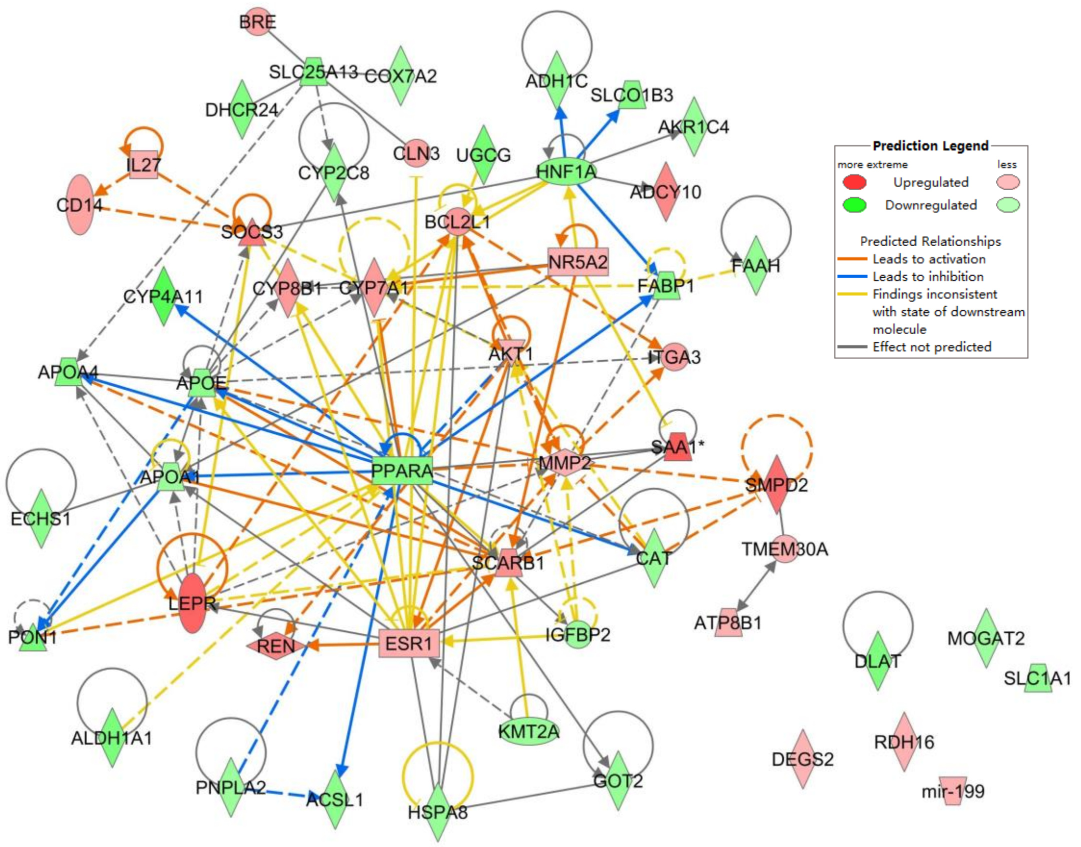

Figure S6 Network interaction of 53 DEGs related with lipid metabolism in IPA. DEGs with red color represent for upregulated while green color represent for downregulated in PreM group compared to PostM group Lines represent known interactions between genes. DEGs: differentially expressed genes. The details of the DEGs refers to Table S5.

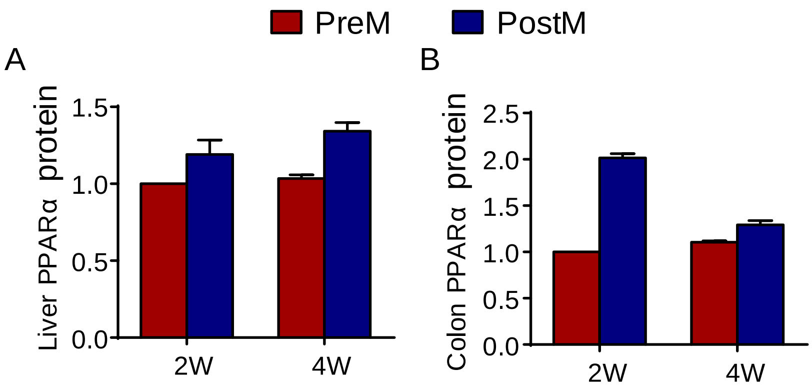

Figure S7 Densitometry analysis of PPARα immunoblots in liver (A) and colon (B) of gnotobiotic mice. The relative expression of PPARα was adjusted by the expression of β-ACTIN as the housekeeping protein. Data are shown as the mean±s.e.m. n=3 for each group. *Pparα*, peroxisome proliferator-activated receptor alpha.

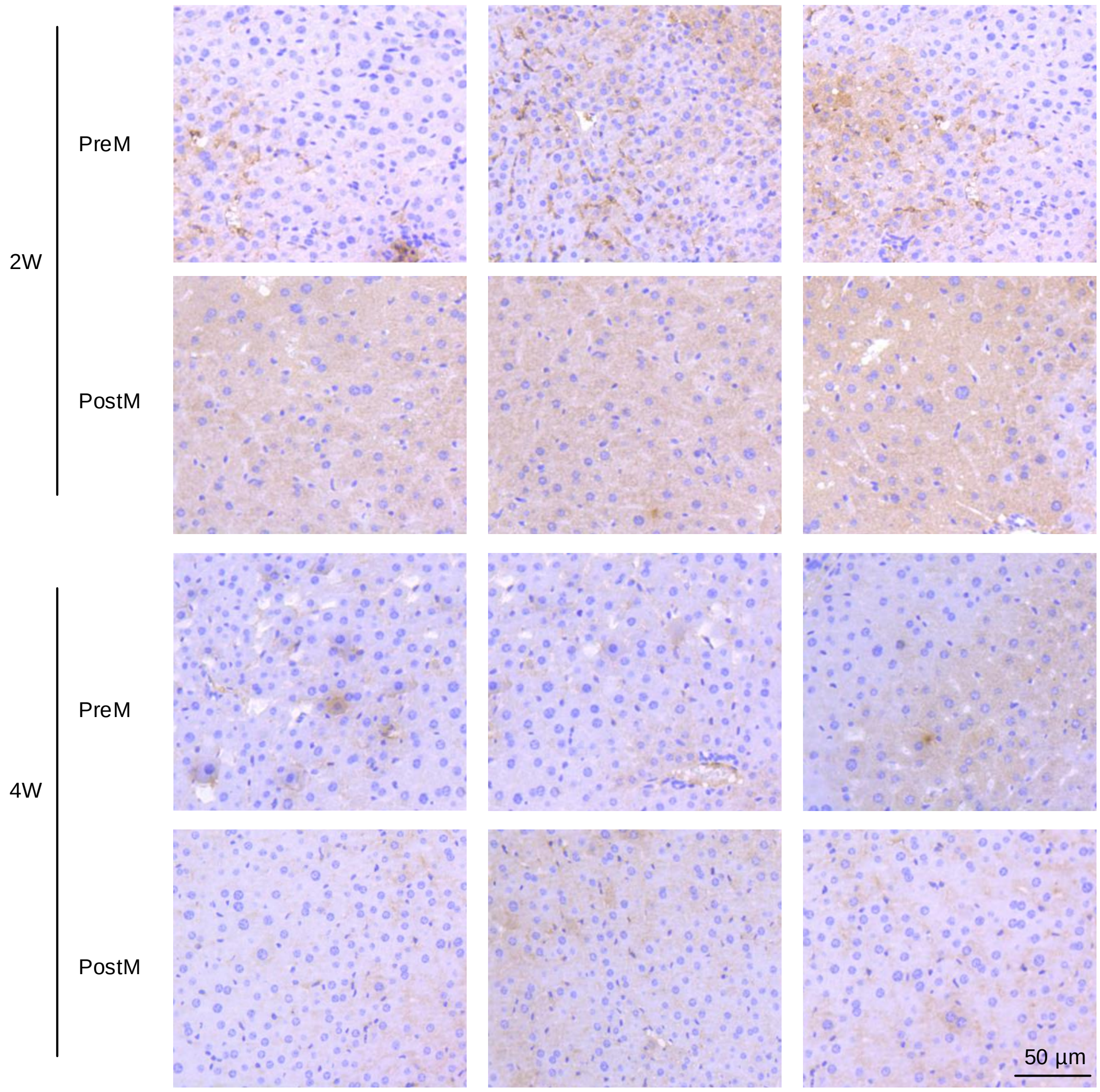

Figure S8 The representative PPARα immunostaining (yellow) sections of liver in gnotobiotic mice (100× magnification). *Pparα*, peroxisome proliferator-activated receptor alpha.

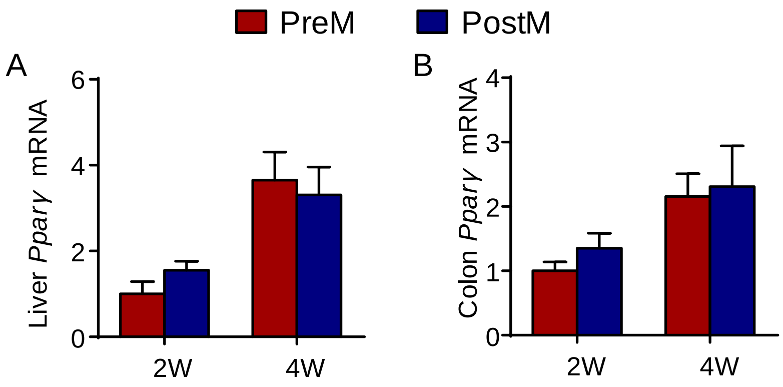

Figure S9 The *pparγ* gene expression in the (a) Liver (b) Colon. The relative expression of *Pparγ* was adjusted with *Gapdh* as the housekeeping gene. Data are shown as the mean±s.e.m. and were analyzed by the Mann-Whitney U test between

groups. n=4 or 5 for each group. *Pparγ*, eroxisome proliferator-activated receptor gamma.

Table S1 Changes of metabolic phenotypes, inflammation indexes and endotoxin load of one genetically obese child (GD58) during 90-day dietary intervention[a].

Measurements	GD58 Pre-intervention	GD58 Post-intervention	Reference range
Body weight (kg)	57.80(100.0%)	49.90(86.3%)	--
BMI(kg m^{-2})	38.64	32.77	[b]
SBP（mm Hg）	139.00	118.00	≤140 for adult
DBP（mm Hg）	95.00	75.00	≤90 for adult
TC（mmol l^{-1}）	5.04	3.55	3.12-5.98
TG（mmol l^{-1}）	1.24	0.72	0.4-1.82
ALT（U/L）	79.00	37.00	0-42
AST（U/L）	53.00	35.00	0-45
Diameter of right lobe of liver(mm)	133	116	--
SAA(μg ml^{-1})	3.42	1.12	--
Leptin(ng ml^{-1})	43.63	18.34	--
LBP(μg ml^{-1})	21.51	17.71	--

[a]This volunteer was included in our previous cohort study and most of his data has been published as an integral previously [1]. PreD, donor pre-intervention; PostD, donor post-intervention.

[b] Standard for overweight and obesity of male at 8 years old: BMI≥18.4 for overweight, BMI≥21.6 for obesity [2].

Abbreviations: AST, aspartate aminotransferase; ALT, alanine aminotransferase; BMI, body mass index; DBP, diastolic blood pressure; LBP, lipopolysaccharide-binding protein; SAA, serum amyloid A protein; TC, total cholesterol; TG, triglyceride

Table S2 OTU table of gut microbiota of donor and recipients.

Table S3 Enriched biological functions in liver between the two groups on the 2nd and 4th week. (NaNs in the table mean not a number)

Table S4 Enriched pathways in liver between the two groups on the 2nd and 4th week.

Table S5 Annotation of the 53 DEGs associated with lipid metabolism.

Table S2 to Table S5 are presented in separate files.

1. Zhang, C., et al., *Dietary Modulation of Gut Microbiota Contributes to Alleviation of Both Genetic and Simple Obesity in Children.* EBioMedicine, 2015. 2(8): p. 968-84.
2. Cole, T.J., et al., *Establishing a standard definition for child overweight and*

obesity worldwide: international survey. Bmj, 2000. 320(7244): p. 1240.
